# Preanalytical Biases in the Measurement of Human Blood Sphingolipids

**DOI:** 10.3390/ijms19051390

**Published:** 2018-05-07

**Authors:** Robert Brunkhorst, Waltraud Pfeilschifter, Sammy Patyna, Stefan Büttner, Timon Eckes, Sandra Trautmann, Dominique Thomas, Josef Pfeilschifter, Alexander Koch

**Affiliations:** 1 Department of Neurology, Goethe University Hospital Frankfurt, 60590 Frankfurt am Main, Germany; waltraud.pfeilschifter@kgu.de; 2 Department of General Pharmacology and Toxicology, Goethe University Hospital Frankfurt, 60590 Frankfurt am Main, Germany; sammy.patyna@googlemail.com (S.P.); eckes@em.uni-frankfurt.de (T.E.); pfeilschifter@em.uni-frankfurt.de (J.P.); koch@med.uni-frankfurt.de (A.K.); 3 Department of Nephrology, Goethe University Hospital Frankfurt, 60590 Frankfurt am Main, Germany; stefan.buettner@kgu.de; 4 Department of Clinical Pharmacology, Goethe University Hospital Frankfurt, 60590 Frankfurt am Main, Germany; labocha@med.uni-frankfurt.de (S.T.); Thomas@med.uni-frankfurt.de (D.T.)

**Keywords:** sphingolipids, sphingosine 1-phosphate, sphinganine 1-phosphate, ceramides, dihydroceramides, blood

## Abstract

Dysregulation of blood sphingolipids is an emerging topic in clinical science. The objective of this study was to determine preanalytical biases that typically occur in clinical and translational studies and that influence measured blood sphingolipid levels. Therefore, we collected blood samples from four healthy male volunteers to investigate the effect of storage conditions (time, temperature, long-term storage, freeze–thaw cycles), blood drawing (venous or arterial sampling, prolonged venous compression), and sample preparation (centrifugation, freezing) on sphingolipid levels measured by LC-MS/MS. Our data show that sphingosine 1-phosphate (S1P) and sphinganine 1-phosphate (SA1P) were upregulated in whole blood samples in a time- and temperature-dependent manner. Increased centrifugation at higher speeds led to lower amounts of S1P and SA1P. All other preanalytical biases did not significantly alter the amounts of S1P and SA1P. Further, in almost all settings, we did not detect differences in (dihydro)ceramide levels. In summary, besides time-, temperature-, and centrifugation-dependent changes in S1P and SA1P levels, sphingolipids in blood remained stable under practically relevant preanalytical conditions.

## 1. Introduction

Besides their role in energy metabolism, several classes of lipids are considered as being “bioactive,” having numerous roles in cell biology, physiology, and disease. Among these lipids, sphingolipids, such as several dihydroceramides (dhCer) and ceramides (Cer), have been increasingly recognized as important mediators of several biological processes [1]. As sphingolipids are not only be detected within cells but also extracellularly, especially in blood, a great interest has been developed regarding their involvement in cell signaling. The abundance of sphingolipids in blood have led to numerous studies not only to define their regulation and function but also to measure their concentration in clinical samples. For example, a decreased serum concentration of sphingosine 1-phosphate (S1P), one of the major sphingolipids in blood, has been shown in atherosclerotic patients suffering from peripheral artery disease and carotid stenosis [2]. In contrast, patients with acute coronary events have been shown to have increased circulating S1P levels [3]. In blood samples from patients with multiple sclerosis, a disease that can be treated by the synthetic sphingolipid analog fingolimod [4], observations pointing toward an involvement of ceramide concentrations have been made [5,6]. Furthermore, in patients with viral hepatitis and hepatocellular carcinoma, extensive biomarker studies with sphingolipids have been performed [7,8]. Recent data indicate an involvement of S1P in sepsis and shock [9]. Tackling the gap between such conclusions and the lack of reference values for S1P in the blood of healthy subjects, Moritz et al. [10] recently provided S1P concentrations of 1339 healthy volunteers. Surprisingly, the range of concentrations was considerably large (95% CI-interval: 0.53–1.24 μmol/L), implying severe difficulties in using S1P as an individual predictor for a certain disease. In that line, control groups of other studies measuring S1P in serum and plasma showed a similar variability [3,9,11]. In light of the published high variability of S1P blood levels in control subjects and the increasing investigation of sphingolipids as a biomarker, we believe that it is important to define the preanalytical factors that could alter sphingolipid concentrations. In general, several possible systematic biases should be considered when conducting biomarker studies [12], especially when collecting under relatively non-standardized conditions in the clinic. Some of these factors have been analyzed before. Scherer et al. [13] investigated whether storage at room temperature up to 24 h influences the amount of S1P and sphinganine 1-phosphate (SA1P) in whole blood samples, as well as in serum and isolated plasma. A similar study has been conducted by Yin et al. [14]. These authors investigated the influence of preanalytical variables on the human blood metabolome and their approach detected a considerable influence of the sampling technique and storage conditions on S1P as well as other parameters. However, no data on other metabolites of the sphingolipid pathway, such as different ceramide species, were provided in those studies. Hammad et al. [15] determined the influence of some preanalytical factors on the whole blood sphingolipidome. They investigated the influence of freeze–thaw cycles, different anticoagulants, the difference between serum and plasma, and the influence of gender and food intake [15].

The goal of this study was to identify and characterize the most relevant factors occurring typically in clinical studies in order to define “standard operating procedures” for measuring sphingolipids in blood. We investigated the sphingolipid profile in human blood, serum, and plasma samples dependent on differences in blood drawing, sample preparation, freezing and storage conditions by high-performance liquid chromatography/tandem mass spectrometry (LC-MS/MS). Our results might be helpful for the design and comparison of future human prospective studies and to look more critically at the conclusions made from results of retrospectively analyzed samples, where the exact sampling conditions were not well defined.

## 2. Results

### 2.1. Characteristics of Study Participants

The characteristics of the four participants are shown in Table 1. The mean age of the study participants was 27.8 ± 6.95 years (mean ± SD) with a body mass index of 24.4 ± 1.85 kg/m^2^ (mean ± SD). Blood laboratory parameters of the volunteers were as followed: cholesterol = 163 ± 31.6, triglycerides = 112 ± 28.5, high-density lipoproteins = 52.5 ± 10.3, low-density lipoproteins = 88.0 ± 32.7 (mg/dL; means ± SD).

### 2.2. Time- and Temperature-Dependent Effects on Sphingolipid Levels in Human Blood Samples

**Figure 1:**
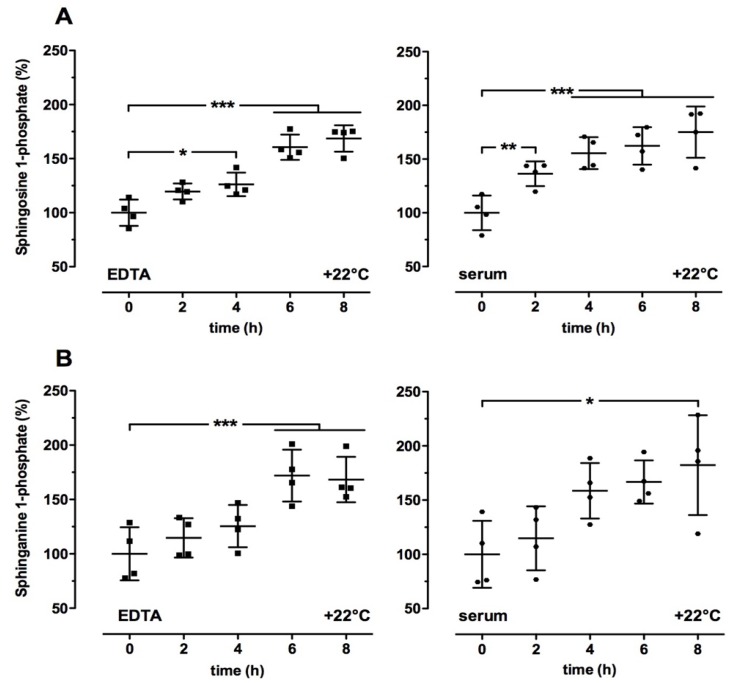
Time-dependent effect on relative sphingosine 1-phosphate (**A**) and sphinganine 1-phosphate (**B**) levels in human blood samples. Whole blood samples (EDTA as anticoagulant or serum) were incubated at +22 °C for the indicated times. Thereafter, plasma and serum were isolated as described in the Material and Methods section. Plasma and serum samples without storage time served as the respective controls (0 h; 100%). After final preparation, all samples were stored at −80 °C until LC-MS/MS analysis. Data are shown as the mean (in %) ± SD (*n* = 4). * *p* < 0.05, *** *p* < 0.001.

**Figure 2:**
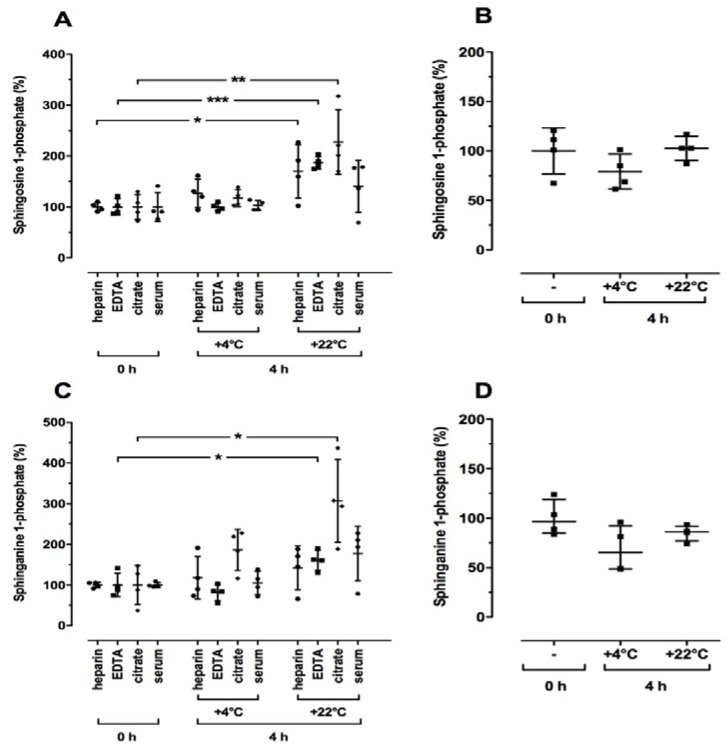
Temperature-dependent effect on relative sphingosine 1-phosphate (**A**,**B**) and sphinganine 1-phosphate (**C**,**D**) levels in human blood samples. Whole blood samples (heparin, EDTA, and citrate as anticoagulants or serum) were incubated for 4 h at the indicated temperatures (**A**,**C**). Thereafter, plasma and serum were isolated as described in the Material and Methods section. (**B**,**D**) Freshly isolated plasma (EDTA as anticoagulant) was incubated for 4 h at the indicated temperatures. Plasma and serum samples without storage time served as the respective controls (0 h; 100%). After final preparation, all samples were stored at −80 °C until LC-MS/MS analysis. Data are shown as the mean (in %) ± SD (*n* = 4). * *p* < 0.05, ** *p* < 0.01, *** *p* < 0.001.

First, we evaluated whether there were changes in sphingolipid levels according to storage time and temperature. We incubated whole blood and serum samples at either +22 °C for different times or at +4 °C and +22 °C for up to 4 h. As shown in Figure 1A,B, S1P and SA1P levels in EDTA blood and serum were significantly increased in a time-dependent manner at +22 °C compared to the respective control (0 h). Further, a 4-h incubation at +22 °C led to elevated S1P and SA1P levels in whole blood samples using heparin, EDTA, and citrate as anticoagulants (Figure 2A,C). In contrast, a 4-h incubation at +4 °C did not significantly alter the relative S1P and SA1P levels in whole blood and serum samples (Figure 2A,C). Incubation of directly isolated plasma samples (EDTA as anticoagulant) for 4 h at +4 °C and +22 °C did not influence S1P and SA1P levels (Figure 2B,D). In order to investigate whether the elevated S1P and SA1P levels shown in Figure 1 and Figure 2 were due to red blood cell damage, we measured several markers for extravascular hemolysis in the whole blood samples stored at +22 °C for up to 8 h or at +4 °C for 4 h. As illustrated in Table S1, we found a time-dependent decrease in relative potassium levels at +22 °C compared to control (0 h, 100%). In contrast, significantly elevated levels of potassium were measured after storage at +4 °C for 4 h compared to the respective control (0 h: 4.39 ± 0.23 mmol/L (mean ± SD) = 100%; Table S1). There were no time-dependent differences in hemoglobin at +22 °C but significantly elevated levels after 4-h storage at +4 °C (Table S1). All other indicators for hemolysis such as bilirubin, aspartate aminotransferase, and lactate dehydrogenase were not altered under either condition compared to the respective control (0 h: total bilirubin: 0.50 ± 0.12 mg/dL, indirect bilirubin: 0.23 ± 0.05 mg/dL, aspartate aminotransferase: 26.8 ± 9.32 IU/L, lactate dehydrogenase: 163 ± 32.1 IU/L (means ± SD) = 100%).

As shown in Table 2, Cer and dhCer levels remained stable in the whole blood and serum samples after incubating at +22 °C for up to 8 h. In whole blood samples with heparin as the anticoagulant, C18Cer was significantly elevated (by 20%) after 4 h incubation at +22 °C compared to directly isolated plasma without storage time (0 h, Table 3). However, there was no further significant alteration in Cer and dhCer levels upon 4 h incubation of either whole blood or directly isolated plasma samples at different temperatures (Table 3 and Table 4).

### 2.3. Effect of Blood Drawing Conditions on Sphingolipid Levels in Human Blood Samples

**Figure 3:**
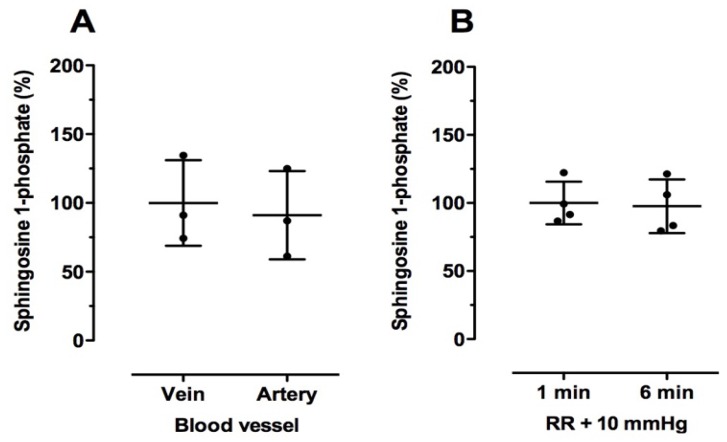

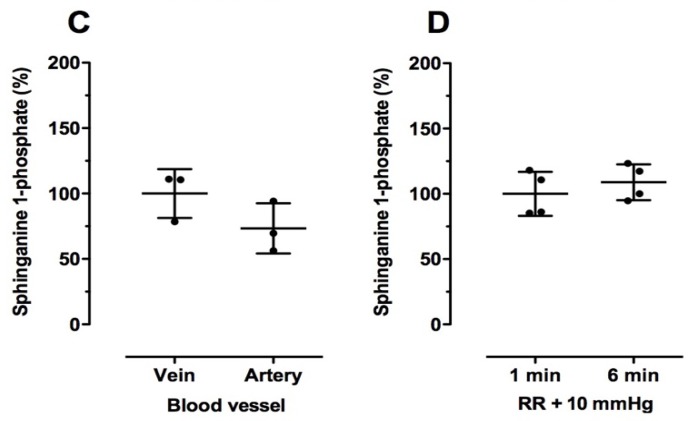
Effect of blood drawing conditions on relative sphingosine 1-phosphate (**A**,**B**) and sphinganine 1-phosphate (**C**,**D**) levels in human blood samples. Blood samples were taken from veins and arteries (**A**,**C**; heparin as anticoagulant) or after 1 min or 6 min RR + 10 mmHg (**B**,**D**; EDTA as anticoagulant). Thereafter, plasma was directly separated as described in the Material and Methods section. Plasma separated from venous blood (**A**,**C**) and after 1 min RR + 10 mmHg (**B**,**D**) served as the respective controls (100%). All plasma samples were stored at −80 °C until LC-MS/MS analysis. Data are shown as mean (in %) ± SD (*n* = 3–4).

Next, we analyzed the effects of the conditions that are typically used in the clinic for blood drawing, which might alter sphingolipid levels in blood. We first evaluated whether there were differences in the sphingolipid content in samples of venous and arterial sources taken from the same volunteers (*n* = 3). By measuring the relative oxygen levels, we confirmed that we had collected venous and arterial blood (venous blood: 100 ± 11.4, arterial blood: 269 ± 62.6 (% of control); mean ± SD; *n* = 3; Paired *t*-test; *p* = 0.04). As shown in Figure 3A,C, there were no significant differences in S1P and SA1P levels in blood samples taken by venous or arterial puncture (heparin as anticoagulant). All Cer and dhCer levels were similar in venous and arterial blood samples (Table 5).

In a second approach, we collected venous blood samples (EDTA as anticoagulant) under normal systolic blood pressure + 10 mmHg for either 1 min or 6 min in order to simulate long-term vein compression by a tourniquet since this can happen when blood sampling proves to be difficult. No changes were observed for S1P or SA1P levels (Figure 3B,D). As shown in Table 5, C24:1Cer levels were significantly lower after 6 min compared to the control (RR + 10 mmHg for 1 min). All other Cer and dhCer levels were not significantly reduced (Table 5).

### 2.4. Effect of Sample Preparation on Sphingolipid Levels in Human Blood Samples

**Figure 4:**
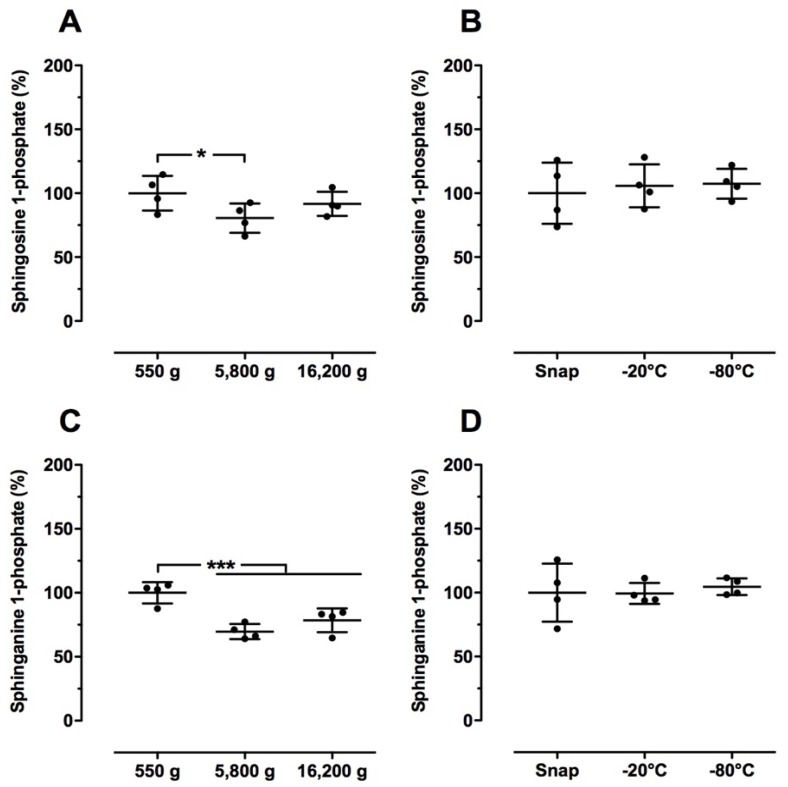
Effect of sample preparation on relative sphingosine 1-phosphate (**A**,**B**) and sphinganine 1-phosphate (**C**,**D**) levels in human blood samples. (**A**,**C**) Plasma was directly separated from blood (EDTA as anticoagulant) by 5 min centrifugation (4 °C) at 550× *g*, 5800× *g* or 16,200× *g*. (**B**,**D**) Plasma was directly separated from blood (EDTA as anticoagulant) as described in the Material and Methods section. Thereafter, plasma samples were either snap frozen in liquid nitrogen or frozen at −20 °C or −80 °C. Plasma separated at 550× *g* (**A**,**C**) and snap frozen (**B**,**D**) served as the respective controls (100%). All plasma samples were stored at −80 °C until LC-MS/MS analysis. Data are shown as mean (in %) ± SD (*n* = 4). * *p* < 0.05, *** *p* < 0.001.

Here, we tested whether different centrifugations steps and freezing conditions influence sphingolipid levels in human blood samples. Interestingly, centrifugation at 5800× *g* and 16,200× *g* significantly lowered the relative S1P and SA1P levels compared to the control (550× *g*), whereas Cer and dhCer levels were not altered (Figure 4A,C, Table 6). No significant changes in sphingolipid levels were observed from the different freezing conditions (snap frozen, −20 °C, −80 °C; Figure 4B,D, Table 6).

### 2.5. Effect of Long-Term Storage Conditions on Sphingolipid Levels in Human Blood Samples

**Figure 5:**
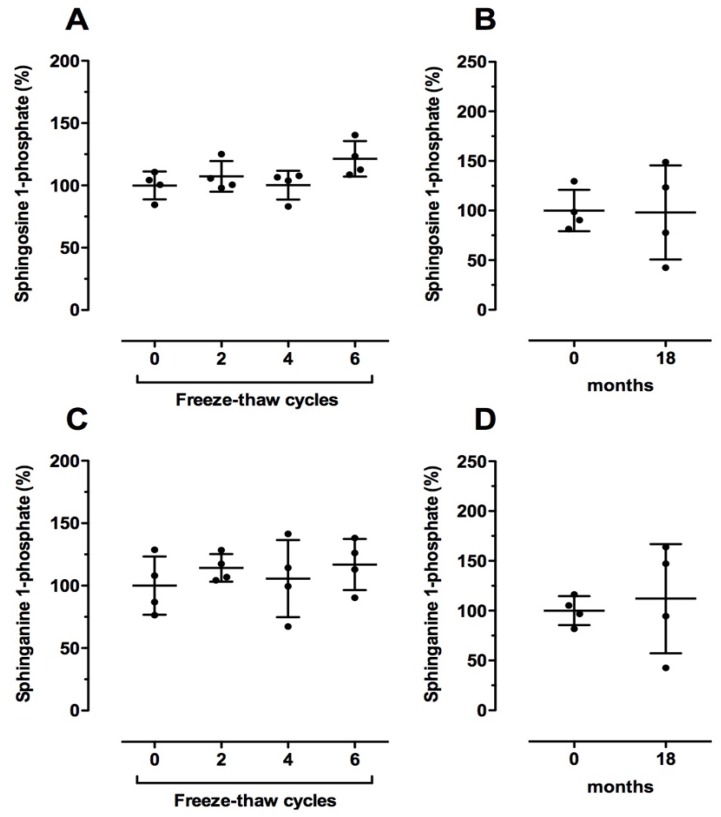
Effect of storage conditions on relative sphingosine 1-phosphate (**A**,**B**) and sphinganine 1-phosphate (**C**,**D**) levels in human blood samples. (**A**–**D**) Plasma was directly separated from blood (EDTA as anticoagulant) as described in the Material and Methods section. (**A**,**C**) Plasma samples were deep frozen at −80 °C for 1 h and completely thawed at +22 °C for 30 min for the indicated number of freeze–thaw cycles. (**B**,**D**) Plasma samples were stored for 18 months at −80 °C before analysis. Plasma without freeze–thaw cycles (**A**,**C**) or long-term storage (**B**,**D**) served as the respective controls (100%). Data are shown as the mean (in %) ± SD (*n* = 4).

First, we checked whether sphingolipid levels were affected by freeze–thaw cycles. For one cycle, plasma samples were frozen at −80 °C for 1 h followed by 30 min thawing at +22 °C. As illustrated in Figure 5A,C and Table 7, S1P, SA1P, Cer, and dhCer levels were not significantly altered by undergoing up to six freeze–thaw cycles.

In order to answer the question of whether sphingolipids remain stable after long-term storage, we analyzed the sphingolipid levels in plasma samples that had been stored for 18 months at −80 °C compared to freshly isolated plasma from the same volunteers (EDTA as anticoagulant). Again, no significant alterations were observed for all analyzed sphingolipids upon long-term storage (Figure 5B,D, Table 7).

## 3. Discussion

We hypothesized that sphingolipid concentrations in blood are subject to different systematic preanalytical factors, thus hampering the generalizability of results of sphingolipid analyses in blood samples under non-controlled conditions. Our data clearly define certain caveats that should be avoided when measuring sphingolipids in blood. In line with previous findings [13,14], we found a time-dependent increase in S1P and SA1P levels in human blood samples that clearly argues for a swift preparation of plasma. Scherer et al. [13] showed this time-dependent effect on S1P and SA1P in whole blood samples stored for 1–24 h at room temperature but not in separated plasma and serum samples. Using a “shot-gun” metabolomics approach, Yin et al. [14] identified S1P as one of the lipids regulated in a time-dependent manner in human blood samples. In addition to these studies, we showed that there is a temperature-dependent effect on S1P and SA1P in whole blood samples. Both sphingolipids remained stable for 4 h at +4 °C but showed elevated levels after 4 h incubation at +22 °C. Compared to the previous publications by Scherer et al. and Yin et al. [13,14], we measured more upstream metabolites of S1P (i.e., ceramides) and SA1P (i.e., dihydroceramides). Interestingly, we did not detect any significant influence of storage time or temperature on the most prominent ceramide and dihydroceramide species in whole blood samples. Further, no significant temperature-dependent effect on any sphingolipid was detected in isolated plasma samples. However, even though the observed changes (Table 2 and Table 3) were not significant in our analysis, they could be relevant in larger sample sizes that are usually analyzed in clinical studies. While this is the main limitation of our proof of concept study, our results may lead to the avoidance of confounders such as time and anticoagulant.

As our study was not designed to explain the effects of temperature and time on sphingolipid concentrations, this needs further investigation. However, some preliminary conclusions could be drawn, e.g., the lack of an increase of these metabolites in isolated plasma suggests an involvement of the platelets or red blood cells. The effects of temperature and time on S1P and SA1P in whole blood samples could have several cellular causes. The most likely explanation is either the activation, destruction, or dysfunction of platelets or red blood cells or a combination of effects, as both cell types contain large amounts of both sphingolipids [16,17]. Concerning the role of blood cell destruction, we evaluated several markers for extracellular hemolysis but found that none of them were increased in a time-dependent manner at +22 °C. In contrast, storage at +4 °C for 4 h led to significantly elevated potassium and hemoglobin levels, but no changes in S1P/SA1P levels were detected. Further, no changes were measured after prolonged venous compression, leading to more hypoxic and hemolytic blood samples. Thus, from these data, we would exclude an enhanced destruction of red blood cells as the cause of the time-dependent elevated S1P/SA1P levels. As reported previously [15], the relevance of platelet activation for S1P/SA1P concentrations in human blood can be seen in the higher concentration after ex vivo coagulation in serum samples. On the other hand, the further increase of S1P in coagulated blood after several hours is unlikely to be explained by increased platelet activation. Most interestingly, the temperature-dependent increase in S1P and SA1P levels in whole blood samples and serum raises some exciting questions about the ex vivo activity of enzymes located in blood cells and involved in the synthesis or degradation of these sphingolipids. In general, S1P and SA1P are synthesized intracellularly from sphingosine via sphingosine kinase (SPHK)-1 and SPHK-2 [16,17]. The degradation of both sphingolipids involves either the dephosphorylation by phosphatases or the irreversible cleavage via the S1P lyase [16,17]. Especially, the lack of S1P lyase in blood cells is thought to be the major reason that platelets and red blood cells serve as the major sources of S1P/SA1P in blood [16,17]. Conversely, the SPHK-1/SPHK-2 double-knockout in embryonic red blood cells reduces circulating S1P to very low levels [18]. Mechanistically, it is likely that with an ongoing energy metabolism of cells, oxygen levels also drop in whole blood samples ex vivo, which is known to increase SPHK activity and S1P concentrations [19]. Even if we did not measure oxygen saturation in our blood samples, we did not measure any differences between arterial and venous blood samples nor after stronger vein compressing. Thus, our data suggest that there is no effect of oxygen desaturation on blood S1P/SA1P levels. Overall, the exact mechanism underlying the observed ex vivo changes at +22 °C in human whole blood samples need to be clarified in future studies.

From the clinical point of view, we showed that most of the relevant preanalytical variables did not influence S1P and SA1P levels in human blood samples. In addition, ceramide and dihydroceramide remained stable in almost all clinically important conditions, such as prolonged tourniquet during blood drawing, long-term storage, and several freeze–thaw cycles. The latter was also investigated by Hammad et al. [15] who checked whether freeze–thaw cycles could influence plasma and serum sphingolipids. Unlike the report by Hammad et al. [15], we did not detect an effect of up to six freeze–thaw cycles on C16Cer or the other sphingolipids analyzed. From our data, it seems to be more important to carefully choose the centrifugation parameters used to isolate plasma from whole blood samples. A lower centrifugation speed could lead to cells (e.g., platelet fragments) remaining in the sample and, thus, higher S1P levels. In addition, Hammad et al. [15] investigated whether differences in the sphingolipid content in blood were not only dependent on the anticoagulant but also on gender and food intake. The authors showed that there was no gender-specific effect on ceramide levels but that there was an increase in some dihydroceramide levels in females compared to male blood samples. Further, they demonstrated that food intake increased the amount of, e.g., C16Cer, C18Cer, and C20Cer in serum samples of both male and female volunteers, whereas S1P and SA1P levels were not affected [15]. Reichel et al. [20] showed that chronic alcohol abuse alters plasma glycerophospholipid as well as several sphingolipid species in human blood. In a larger cohort of healthy participants, it was shown that serum S1P levels were not affected by age, gender, BMI, or smoking status [10,21]. Baranowski et al. [22] showed that endurance training increases plasma S1P levels, most likely via elevated HDL-bound S1P probably due to enhanced SPHK activation and S1P release from red blood cells [23]. Thus, we should also keep in mind lifestyle related confounders when drawing conclusions from clinical datasets.

Taken together, our study gives important implications for future biomarker studies concerning the stability of sphingolipids. From our data, several recommendations can be extracted: Ceramides are surprisingly stable under the various conditions tested here. Besides this important implication for the reliability of studies about ceramides, we recommend especial caution if S1P or SA1P are in focus; study groups should avoid long processing times of blood samples, the use of different anticoagulants, and centrifugation steps. On the other hand, samples can be drawn from different types of vessels without any restrictions regarding vein compression time. As soon as plasma is isolated, also S1P and SA1P remain stable under various storage conditions.

## 4. Materials and Methods

### 4.1. Blood Sampling

If not otherwise indicated, venous blood (single puncture per day and experiment of venae brachiales, different anticoagulants (EDTA, heparin, and citrate) and serum, 1 mL each) was used from four healthy non-fasting male volunteers. Plasma and serum (after coagulation) separation was performed by two-times centrifugation at 550× *g* for 5 min (4 °C). Thereafter, 20 µL aliquots were stored immediately at −80 °C until lipid extraction and LC-MS/MS measurement. The study was performed in accordance with the Declaration of Helsinki. Blood drawing was approved by the local ethics committee. All volunteers had signed a written informed consent.

### 4.2. Experimental Setup

The following experiments were performed: (1) Whole blood samples (heparin, EDTA, citrate, and serum) were incubated at +4 °C and +22 °C for 4 h or at +22 °C for 0–8 h; (2) Separated plasma samples (EDTA) were incubated at +4 °C and +22 °C for 4 h. In both cases, immediately separated plasma and serum samples without incubation served as controls (0 h); (3) Venous (venae brachiales) and arterial (arteria radialis) blood samples (heparin) were collected and plasma was immediately separated; (4) Blood samples (EDTA) were collected under normal systolic blood pressure (RR) with compression +10 mmHg for either 1 min or 6 min to stimulate prolonged venous compression as it is often necessary when blood sampling proves to be difficult. Thereafter, plasma was immediately separated; (5) Plasma samples were separated from blood (EDTA) by 5 min centrifugation (+4 °C) at 550× *g*, 5800× *g*, or 16,200× *g*; (6) Separated plasma samples (EDTA) were either snap-frozen in liquid nitrogen or frozen at −20 °C and −80 °C. After freezing, all aliquots were stored at −80 °C until lipid extraction; (7) Separated plasma (EDTA) was frozen at −80 °C for 1 h and completely thawed at +22 °C for 30 min. Freeze–thaw cycles were repeated 2–6 times. Plasma without additional freezing/thawing cycles served as the respective control; (8) Separated plasma (EDTA) was stored for 18 months at −80 °C. Freshly isolated plasma without long-term storage served as the respective control. After one-time thawing, aliquots were prepared for lipid extraction.

### 4.3. LC-MS/MS Analysis

For lipid extraction, 10 µL human plasma and/or serum were mixed with 150 µL water, 150 µL extraction buffer (citric acid 30 mM, disodium hydrogen phosphate 40 mM), and 20 µL of the internal standard solution containing sphingosine-d7, sphinganine-d7 (200 ng/mL each), sphingosine 1-phosphate-d7, C17:0 Cer, C16:0 Cer-d31, C18:0 Cer-d3, C18:0 dhCer-d3 (all Avanti Polar Lipids, Alabaster, AL, USA), and C24:0 Cer-d4 (Chiroblock GmbH, Bitterfeld-Wolfen, Germany) (400 ng/mL each). The mixture was extracted once with 1000 µL methanol/chloroform/hydrochloric acid (15:83:2, *v*/*v*/*v*). The lower organic phase was evaporated at +45 °C under a gentle stream of nitrogen and reconstituted in 200 µL of tetrahydrofuran/water (9:1, *v*/*v*) with 0.2% formic acid and 10 mM ammonium formate. Afterward, amounts of sphingolipids were analyzed by liquid chromatography coupled to tandem mass spectrometry (LC-MS/MS). An Agilent 1100 series binary pump (Agilent Technologies, Waldbronn, Germany) equipped with a Luna C8 column (150 mm × 2 mm ID, 3 μm particle size, 100 Å pore size; Phenomenex, Aschaffenburg, Germany) was used for chromatographic separation. The column temperature was 35 °C. The HPLC mobile phases consisted of water with 0.2% formic acid and 2 mM ammonium formate (mobile phase A) and acetonitrile/isopropanol/acetone (50:30:20, *v*/*v*/*v*) with 0.2% formic acid (mobile phase B). For separation, a gradient program was used at a flow rate of 0.3 mL/min. The initial buffer composition 55% (A)/45% (B) was held for 0.7 min and then within 4.0 min linearly changed to 0% (A)/100% (B) and held for 13.3 min. Subsequently, the composition was linearly changed within 1.0 min to 75% (A)/25% (B) and then held for another 2.0 min. The total running time was 21 min and the injection volume was 15 μL. To improve ionization, acetonitrile with 0.1% formic acid was infused post-column using an isocratic pump at a flow rate of 0.15 mL/min. After every sample, sample solvent was injected for washing the column with a 12 min run. The MS/MS analyses were performed using a triple quadrupole mass spectrometer API4000 (Sciex, Darmstadt, Germany) equipped with a Turbo V Ion Source operating in positive electrospray ionization mode. The MS parameters were set as follows: Ionspray voltage 5500 V, ion source temperature +500 °C, curtain gas 30 psi, collision gas 12 psi, nebulizer gas 40 psi, and heating gas 60 psi. The analysis was done in Multiple Reaction Monitoring (MRM) mode. Data acquisition was done using Analyst Software V 1.6 and quantification was performed with MultiQuant Software V 3.0 (both Sciex, Darmstadt, Germany), employing the internal standard method (isotope dilution mass spectrometry). Variations in accuracy of the calibration standards were less than 15% over the whole range of calibration, except for the lower limit of quantification where a variation in accuracy of 20% was accepted.

### 4.4. Statistical Analysis

Statistical analyses were performed with GraphPad Prism (v5.01; GraphPad Software Inc., San Diego, CA, USA). Significant differences between two groups were evaluated by paired *t*-test and between more than two groups by repeated ANOVA followed by Bonferroni post hoc test. Differences with *p* < 0.05 were considered to be significant. All data are shown as mean (in %) ± standard deviation (SD). Analysis were performed on relative values. For better comparability, time- and temperature-dependent effects on absolute SL concentrations are examplified in Table S2.

## Figures and Tables

**Table 1:** Individual characteristics and blood laboratory parameters of study participants.

Participant	Age (years)	BMI (kg/m^2^)	Triglycerides (mg/dL)	Cholesterol (mg/dL)	HDL (mg/dL)	LDL (mg/dL)
1	35	24.7	143	185	40	117
2	20	26.6	80	153	48	89
3	32	24.3	127	190	61	104
4	24	22.1	97	122	61	42

Abbreviations: BMI, body mass index; HDL, high-density lipoproteins; LDL, low-density lipoproteins.

**Table 2:** Time-dependent effect on relative sphingolipid levels in human blood samples.

	+22 °C				
	0 h	2 h	4 h	6 h	8 h
**EDTA**					
C16Cer	100 ± 68.4	66.9 ± 35.4	96.3 ± 76.4	58.8 ± 12.0	65.7 ± 21.9
C18Cer	100 ± 32.0	86.1 ± 62.5	98.8 ± 44.1	69.8 ± 29.3	88.5 ± 42.7
C20Cer	100 ± 17.9	89.6 ± 33.8	105 ± 56.2	96.3 ± 54.9	98.7 ± 52.0
C24Cer	100 ± 17.9	92.9 ± 26.0	92.7 ± 27.0	92.0 ± 23.5	92.7 ± 24.3
C24:1Cer	100 ± 11.4	95.8 ± 35.3	98.7 ± 12.1	100 ± 34.1	99.1 ± 37.1
C24dhCer	100 ± 20.6	91.6 ± 20.2	103 ± 18.7	89.2 ± 16.0	101 ± 14.6
C24:1dhCer	100 ± 22.6	104 ± 26.4	103 ± 27.8	112 ± 43.0	108 ± 34.7
**Serum**					
C16Cer	100 ± 62.5	80.9 ± 45.8	62.9 ± 27.8	55.8 ± 30.2	102 ± 84.0
C18Cer	100 ± 111	82.3 ± 74.4	55.5 ± 25.0	42.3 ± 12.5	65.9 ± 17.0
C20Cer	100 ± 19.6	101 ± 79.6	85.7 ± 34.2	82.1 ± 18.0	105 ± 50.4
C24Cer	100 ± 24.6	113 ± 21.9	105 ± 25.4	99.6 ± 30.3	106 ± 23.6
C24:1Cer	100 ± 19.3	104 ± 28.3	88.8 ± 34.1	82.0 ± 30.1	110 ± 41.5
C24dhCer	100 ± 27.7	102 ± 28.8	102 ± 29.6	103 ± 47.7	101 ± 32.2
C24:1dhCer	100 ± 16.7	112 ± 22.0	97.9 ± 29.4	94.3 ± 31.0	117 ± 40.4

Mean (in %) ± SD; *n* = 4; Repeated ANOVA (Bonferroni post test).

**Table 3:** Temperature-dependent effect on relative sphingolipid levels in human blood samples.

	0 h	4 h	
	-	+4 °C	+22 °C
**Heparin**			
C16Cer	100 ± 34.5	88.3 ± 41.6	91.1 ± 23.5
C18Cer	100 ± 42.7	103 ± 44.4	120 ± 34.9 *
C20Cer	100 ± 40.6	106 ± 46.5	91.5 ± 35.0
C24Cer	100 ± 23.9	102 ± 22.8	107 ± 25.5
C24:1Cer	100 ± 26.5	95.7 ± 17.8	105 ± 32.1
C24dhCer	100 ± 37.7	94.1 ± 26.1	94.6 ± 35.6
C24:1dhCer	100 ± 27.7	91.2 ± 24.5	100 ± 34.5
**EDTA**			
C16Cer	100 ± 23.0	107 ± 38.6	91.9 ± 24.3
C18Cer	100 ± 49.1	99.1 ± 44.4	110 ± 44.1
C20Cer	100 ± 66.8	90.7 ± 32.2	102 ± 43.4
C24Cer	100 ± 25.9	102 ± 24.9	105 ± 25.8
C24:1Cer	100 ± 32.0	101 ± 25.7	106 ± 25.5
C24dhCer	100 ± 28.9	117 ± 36.2	98.1 ± 21.9
C24:1dhCer	100 ± 32.2	94.5 ± 23.5	103 ± 25.9
**Citrate**			
C16Cer	100 ± 29.2	110 ± 21.4	102 ± 15.4
C18Cer	100 ± 50.1	104 ± 37.3	92.3 ± 37.6
C20Cer	100 ± 51.7	98.9 ± 47.5	101 ± 41.3
C24Cer	100 ± 26.0	105 ± 24.4	104 ± 24.4
C24:1Cer	100 ± 19.8	105 ± 16.7	99.5 ± 24.3
C24dhCer	100 ± 35.9	106 ± 26.8	117 ± 38.8
C24:1dhCer	100 ± 26.6	102 ± 18.2	100 ± 29.3
**Serum**			
C16Cer	100 ± 45.4	106 ± 46.5	116 ± 43.3
C18Cer	100 ± 42.9	102 ± 43.9	101 ± 51.1
C20Cer	100 ± 40.8	98.1 ± 34.9	93.0 ± 34.2
C24Cer	100 ± 26.0	99.8 ± 21.6	99.9 ± 28.1
C24:1Cer	100 ± 22.3	100 ± 13.2	109 ± 36.9
C24dhCer	100 ± 39.4	102 ± 24.6	107 ± 50.8
C24:1dhCer	100 ± 31.6	90.8 ± 14.0	104 ± 34.1

Mean (in %) ± SD; *n* = 4; Repeated ANOVA (Bonferroni post test); * *p* < 0.05, compared to 0 h.

**Table 4:** Temperature-dependent effect on relative sphingolipid levels in freshly isolated human plasma.

	0 h	4 h	
	-	+4 °C	+22 °C
C16Cer	100 ± 31.1	113 ± 36.8	115 ± 44.2
C18Cer	100 ± 43.3	114 ± 39.2	98.5 ± 37.0
C20Cer	100 ± 52.3	110 ± 68.7	74.0 ± 30.9
C24Cer	100 ± 30.7	99.7 ± 20.7	95.3 ± 25.0
C24:1Cer	100 ± 40.7	110 ± 34.1	113 ± 52.4
C24dhCer	100 ± 29.7	99.5 ± 29.3	91.7 ± 25.2
C24:1dhCer	100 ± 41.9	106 ± 28.9	95.5 ± 35.5

Mean (in %) ± SD; EDTA; *n* = 4; Repeated ANOVA (Bonferroni post test).

**Table 5:** Effect of blood drawing conditions on relative sphingolipid levels in human blood samples.

	Blood vessel		RR + 10 mmHg	
	Vein	Artery	1 min	6 min
C16Cer	100 ± 10.2	106 ± 23.2	100 ± 20.1	93.4 ± 20.8
C18Cer	100 ± 10.4	108 ± 21.3	100 ± 25.6	93.5 ± 30.1
C20Cer	100 ± 7.56	116 ± 4.92	100 ± 41.2	96.4 ± 35.6
C24Cer	100 ± 10.5	76.1 ± 10.0	100 ± 30.4	95.9 ± 22.7
C24:1Cer	100 ± 11.0	109 ± 11.0	100 ± 20.6	93.2 ± 22.4 *
C24dhCer	100 ± 32.3	124 ± 27.0	100 ± 35.5	94.9 ± 32.2
C24:1dhCer	100 ± 20.7	89.7 ± 13.6	100 ± 27.0	95.0 ± 22.1

Mean (in %) ± SD; Blood vessel: Heparin, *n* = 3 (C20Cer: *n* = 2); RR + 10 mmHg: EDTA, *n* = 4; Paired *t* test; * *p* < 0.05.

**Table 6:** Effect of sample preparation on relative sphingolipid levels in human blood samples.

	Centrifugation			Freezing		
	550× *g*	5800× *g*	16,200× *g*	Snap	−20 °C	−80 °C
C16Cer	100 ± 15.9	95.2 ± 17.5	93.6 ± 22.8	100 ± 36.0	112 ± 22.6	105 ± 23.0
C18Cer	100 ± 33.9	105 ± 50.0	96.7 ± 42.4	100 ± 50.2	108 ± 43.1	111 ± 41.1
C20Cer	100 ± 35.7	94.1 ± 48.3	116 ± 4.92	100 ± 59.5	104 ± 38.0	104 ± 52.8
C24Cer	100 ± 19.7	97.2 ± 24.4	92.8 ± 25.1	100 ± 37.0	101 ± 20.0	99.7 ± 24.1
C24:1Cer	100 ± 32.9	90.9 ± 25.9	89.9 ± 24.4	100 ± 36.4	103 ± 24.2	102 ± 29.0
C24dhCer	100 ± 17.0	92.6 ± 23.6	87.7 ± 24.1	100 ± 31.1	103 ± 21.9	103 ± 25.7
C24:1dhCer	100 ± 11.4	93.8 ± 13.2	91.0 ± 15.8	100 ± 22.1	103 ± 17.4	101 ± 14.0

Mean (in %) ± SD; EDTA; *n* = 4; Repeated ANOVA (Bonferroni post test).

**Table 7:** Effect of storage conditions on relative sphingolipid levels in human blood samples.

	Freeze-Thaw Cycles				Storage (Months)	
	0	2	4	6	0	18
C16Cer	100 ± 13.8	105 ± 17.3	98.9 ± 23.6	107 ± 18.8	100 ± 35.3	92.6 ± 20.6
C18Cer	100 ± 24.7	103 ± 24.3	93.9 ± 21.3	106 ± 31.0	100 ± 26.5	117 ± 44.8
C20Cer	100 ± 21.4	106 ± 26.5	98.3 ± 17.5	109 ± 24.5	100 ± 27.9	89.9 ± 30.6
C24Cer	100 ± 33.1	108 ± 34.9	97.2 ± 34.3	110 ± 32.6	100 ± 28.7	96.9 ± 38.0
C24:1Cer	100 ± 27.1	106 ± 26.1	97.1 ± 21.3	108 ± 28.4	100 ± 21.6	98.6 ± 30.4
C24dhCer	100 ± 28.0	100 ± 29.3	93.2 ± 33.7	106 ± 32.2	100 ± 35.4	77.0 ± 37.3
C24:1dhCer	100 ± 26.0	105 ± 26.0	99.3 ± 28.9	113 ± 28.9	100 ± 25.0	97.1 ± 35.8

Mean (in %) ± SD; EDTA; *n* = 4 (Storage: C18Cer: *n* = 3); Repeated ANOVA (Bonferroni post test) or Paired *t* test.

## Supplementary Materials

Supplementary materials can be found at http://www.mdpi.com/1422-0067/19/5/1390/s1.

## Abbreviations

Cer	ceramide
dhCer	dihydroceramide
LC-MS/MS	liquid chromatography-tandem mass spectrometry
SA1P	sphinganine 1-phosphate
S1P	sphingosine 1-phosphate
SPHK	sphingosine kinase
