# Supplementary material for: Preanalytical Biases in the Measurement of Human Blood Sphingolipids
Source: Int J Mol Sci. 2018 May 7;19(5):1390. doi: 10.3390/ijms19051390 (PMC5983773; doi:10.3390/ijms19051390)
Supplement: Supplementary file 1 [file ijms-19-01390-s001.pdf]

Supplementary Materials: Preanalytical Biases in the Measurement of Human Blood Sphingolipids

Robert Brunkhorst, Waltraud Pfeilschifter, Sammy Patyna, Stefan Büttner, Timon Eckes, Sandra Trautmann, Dominique Thomas, Josef Pfeilschifter and Alexander Koch

1. Supplement

1.1. Material and Methods

1.1.1. Measurement of Markers for Extracellular Hemolysis

Potassium, bilirubin, aspartate aminotransferase (AST), lactate dehydrogenase (LDH) levels in human serum samples were routinely measured at the central laboratory of the Goethe University Hospital Frankfurt. Hemoglobin levels in human plasma samples (EDTA) were colorimetric determined at 540 nm using Drabkin's reagent.

1.2. Tables

Table S1. Time- and temperature-dependent effect on relative levels of marker for extravascular hemolysis in human blood samples.

	**+22 °C**					**+4 °C**
	**0 h**	**2 h**	**4 h**	**6 h**	**8 h**	**4 h**
Potassium	100 ± 5.12	98.3 ± 5.22	96.3 ± 5.02 *	96.0 ± 6.00 **	95.3 ± 6.72 **	111 ± 4.98 ***
Bilirubin (total)	100 ± 23.1	100 ± 36.5	105 ± 30.0	105 ± 30.0	95.0 ± 19.2	90.0 ± 25.8
Bilirubin (ind.)	100 ± 22.2	111 ± 25.7	111 ± 25.7	111 ± 25.7	100 ± 22.2	100 ± 22.2
AST	100 ± 34.9	97.2 ± 26.1	98.1 ± 31.6	106 ± 34.7	101 ± 33.0	99.1 ± 28.9
LDH	100 ± 19.7	101 ± 16.9	104 ± 9.84	104 ± 15.8	96.9 ± 15.9	97.2 ± 14.6
Hemoglobin	100 ± 16.3	96.2 ± 18.0	110 ± 24.4	92.3 ± 11.3	108 ± 25.3	148 ± 39.4 **

Mean (in %) ± SD; $n = 4$; Repeated ANOVA (Bonferroni post test); * $p < 0.05$, ** $p < 0.01$, *** $p < 0.001$ compared to 0 h (+22 °C). Abbreviations: AST, aspartate aminotransferase; LDH, lactate dehydrogenase.

Table S2. Baseline sphingolipid concentrations.

	**Plasma (ETDA)**	**Serum**
C16Cer	120 ± 79.5	99.6 ± 82.8
C18Cer	40.2 ± 17.1	35.0 ± 10.2
C20Cer	68.3 ± 20.2	73.6 ± 36.2
C24Cer	1512 ± 327	1476 ± 420
C24:1Cer	424 ± 57.0	411 ± 113
C24dhCer	90.3 ± 24.6	81.6 ± 20.3
C24:1dhCer	58.3 ± 15.6	56.8 ± 17.1
S1P	240 ± 29.0	301 ± 76.1
SA1P	47.0 ± 11.5	52.0 ± 16.1

Mean ± SD (ng/mL); $n = 4$.
